# Differential Gene Susceptibility to Sperm DNA Damage: Analysis of Developmental Key Genes in Trout

**DOI:** 10.1371/journal.pone.0114161

**Published:** 2014-12-05

**Authors:** Silvia González-Rojo, Cristina Fernández-Díez, Susana M. Guerra, Vanesa Robles, Maria Paz Herraez

**Affiliations:** Department of Molecular Biology, University of León, León, Spain; Clermont-Ferrand Univ., France

## Abstract

Sperm chromatin in mammals is packaged in different blocks associated to protamines (PDNA), histones (HDNA), or nuclear matrix proteins. Differential packaging has been related to early or late transcription and also to differential susceptibility to genotoxic damage. Genes located in the more accessible HDNA could be more susceptible to injuries than those located in PDNA, being potential biomarkers of paternal DNA damage. Fish sperm chromatin organization is much diversified, some species lacking protamines and some others totally depleted of histones. Analyzing genotoxic damage in a species homogeneously compacted with some sperm nuclear basic protein type, could help in deciphering the clues of differential susceptibility to damage. In the present study we analyzed in rainbow trout the differential susceptibility of nine genes to UV irradiation and H_2_O_2_ treatment. The absence of histones in the sperm nuclei was confirmed by Western blot. The chromatin fractionation in sensitive and resistant regions to *PvuII* (presumably HDNA-like and PDNA-like, respectively) revealed that the nine genes locate in the same resistant region. The number of lesions promoted was quantified using a qPCR approach. Location of 8-hydroxyguanosine (8-OHdG) was analyzed by immunocytochemistry and confocal microscopy. UV irradiation promoted similar number of lesions in all the analyzed genes and a homogenous distribution of 8-OHdG within the nuclei. 8-OHdG was located in the peripheral area of the nucleus after H_2_O_2_ treatment, which promoted a significantly higher number of lesions in developmental-related genes (8.76–10.95 lesions/10 kb) than in rDNA genes (1.05–1.67 lesions/10 kb). We showed for the first time, that differential susceptibility to damage is dependent on the genotoxic mechanism and relies on positional differences between genes. Sensitive genes were also analyzed in cryopreserved sperm showing a lower number of lesions than the previous treatments and a predominant peripheral distribution of oxidative damage (8-OHdG).

## Introduction

Sperm chromatin integrity has recently been considered as a key factor in the control of embryo development and an evident relationship has been established between chromatin structure stability and seminal fertility [1]. Currently, sperm chromatin is considered to be even more important during early embryo development than during the fertilization process [2].

In most vertebrates, sperm nuclei show DNA compacted with protamines as a strategy for protecting genetic material. During mammalian spermatogenesis, most histones are replaced by transition proteins and then by protamines [3]. This process is not homogeneous, the chromatin being packaged in three different arrangements: i) DNA linked to histones in a nucleosomal organization (HDNA), which represents 1–15% of the chromatin; ii) DNA bound to protamines (PDNA), which forms the characteristic toroids in the spermatozoa nuclei and iii) a small fraction of DNA attached to the sperm nuclear matrix, both between the nucleosomes or the toroids [4].

Differential packaging of paternal genes has been described in mammalian spermatozoa and has been related to their early or late transcription during embryo development. Recent studies highlight that genes for early development with a contribution to totipotency, developmental decisions and imprinting patterns, are associated preferentially to histones, located in nucleosomes and associated to particular histone modifications and to hypomethylated DNA regions, being more accessible for early transcription [4]–[7]. These special characteristics have been particularly noticed in human sperm across the HOX loci [5]; whereas genes such as the ribosomal RNA (rDNA), non-essential for developmental decisions, are known to show hypermethylated DNA [8]. The more relaxed packaging could render developmental genes more exposed to damaging agents and consequently more susceptible to suffering injuries. This hypothesis was strengthened by the study of Noblanc *et al.*, [9], who promoted oxidative damage in wild type mouse by treatment with H_2_O_2_ and also analyzed the oxidative lesions promoted *in vivo* in the *gpx5^-^/^-^* mutant, lacking glutathione peroxidase 5. In this study 8-hydroxyguanosine (8-OHdG) located in the peripheral and basal regions of the mouse sperm nucleus, colocalizing with the H3 histone and TOPO2β at the histone-rich and nuclear matrix-attached domains, but not with protamine 1 (PRM1).

Methods traditionally used for the evaluation of chromatin integrity, such as the comet assay, SCSA or TUNEL, analyze different aspects of the status of the whole nuclear genome but are not sensitive enough to detect damage in key genes. Accurate procedures have recently been developed to quantify the number of lesions in specific genes using quantitative PCR (qPCR) [10]. The analysis is based on the capacity of certain DNA lesions (abasic sites, cross-linking, double lesions, modification of nitrogenous bases, strand breakages, DNA fragmentation) to delay and block the polymerase advance in template DNA, causing a decrease in the number of amplified products and a delay in the threshold cycle (Ct). The treatment of the results yields the increase in the number of lesions respect to the basal conditions. This approach, applied to the study of DNA damage during human sperm freezing, allowed our teamto detect a significant number of lesions in key genes for fertilization and early embryo development in normozoospermic donors [11] and to quantify lesions in specific genes in primordial germ cells (PGCs) from zebrafish [12].

Fish spermatozoa display a more diversified pattern of chromatin condensation, the evolution of sperm nuclear basic proteins (SNBPs) being a matter of study [13]–[16]. In some species, a total replacement of histones by protamines has been reported, for example, in *Oncorhynchus keta* [15]; whereas in others, DNA is associated to somatic-like histones forming nucleosomes, as described in zebrafish or seabream sperm [7], [17]. A third group is characterized by the presence of proteins with an intermediate composition between protamines and histones, the protamine-like proteins and red mullet (*Mullus surmuletus*) sperm are an example [18]. Considering this varied landscape, the regulation of early or late transcription should rely on factors other than the presence of HDNA and PDNA, and susceptibility to damage could be similar among genes in those species presenting only one type of nuclear basic proteins. In fact, previous studies by our group revealed few lesions in two nuclear genes related to growth and development, *Igf1* and *Gh*, after cryopreservation of seabream sperm [19], whose DNA was homogeneously compacted with histones [17]. The analysis of zebrafish sperm epigenome [7], lacking protamines, also revealed the presence of blocks of chromatin independent of the nuclear basic proteins, defined by the low DNA methylation and the presence of multivalent histone modifications. These chromatin blocks pack genes for embryo development, including a similar set of genes than those identified in human and mice in nucleosomal regions, with a similar epigenetic pattern [7]. Nevertheless, the candidate gene approach for the evaluation of genotoxic damage has never been used -either in mammals or in fish- comparing genes differentially packaged in the sperm chromatin.

In this respect fish can be particularly challenging because of the fish-specific round of whole genome duplication that takes place during fish radiation [20]. This genome duplication generated a high number of paralogous, which underwent different evolutive processes, and were differentially expressed [21]. The number of paralogous is particularly high in salmonid fish, which have undergone an extra round of genome duplication [22]. As an example, the *Hox* genes are organized in 4 clusters with 39 genes in amniotes, 7 clusters with 45–49 genes in most ray-finned fish and 13 clusters with 118 genes in *Salmo salar* [23]. Taken the different roles adopted by paralogous genes, and their different regulation and expression patterns, this could represent an additional difficulty when exploring the candidate gene approach in fish, particularly in salmonids.

In this context our objective was to analyze the sensitivity of genes, presumably differentially packaged, to UV irradiation and oxidative stress by treatment with H_2_O_2_ in the sperm of rainbow trout (*Oncorhynchus mykiss*), a salmonid whose chromatin structure is controversial. According to Avranova *et al.*, [24], trout sperm was supposed to contain protamines and non-protamine proteins which showed the characteristics of the core histones. In contrast, Saperas and colleagues [18] described nuclear sperm proteins of rainbow trout as protamines. Moreover, the analysis of damages promoted by cryopreservation in the most susceptible genes to the genotoxicants could help us to identify the best candidate genes to be used as sentinels of DNA damage.

## Materials and Methods

### Reagents

All media components were purchased from Sigma (Sigma-Aldrich Spain, Madrid) except when otherwise stated.

### Animals

Sperm samples were obtained from sex-reversed ripe rainbow trout (*Oncorhynchus mykiss*) females (neomales), kept under natural photoperiod in Ovapiscis S.A. (Lugo, Spain). Animal handling was carried out in accordance with the Guidelines of the European Union Council (86/609/EU, modified by 2003/65/CEE), following Spanish regulations (RD 1201/2005, abrogated by RD 53/2013) for the use of laboratory animals, and were approved by the Committee on the Ethics of Animal Experiments of University of León (Permit Number: 15–2011). Animals were sacrificed using the concussion method by qualified personal and all efforts were made to minimize suffering.

### Gamete collection

Seven neomales were slaughtered, the testicles were extracted and the main blood vessels removed. Sperm was obtained with a scalpel by an incision in the testicle, diluted 1∶10 in Storfish commercial maturation medium (IMV, France) and stored for 2 h at 4°C with aeration until the arrival at the facilities of the University of León.

### Experimental procedure

Each milt sample (n = 7) was divided into five aliquots: one of them served as a control and the others were treated with different damaging agents (UV irradiation, hydrogen peroxide treatment and cryopreservation according to two different protocols). During the entire procedure, the sperm was kept at 4°C. In order to carry out the UV and H_2_O_2_ treatments, sperm samples from each male were diluted 40-fold with SFMM (Seminal Fluid Mimicking Medium) (110 mM NaCl, 28.18 mM KCl, 1.22 mM MgSO_4_·7H_2_O, 1.77 mM CaCl_2_·2H_2_O, 10.05 mM bicine, 9.9 mM HEPES, pH 7.4). One aliquot was then transferred to a Petri dish and subjected to UV irradiation (254 nm) with an intensity of 400 µW/cm^2^ (Vilber, Germany) for 10 min at a distance of 15 cm from the lamp. Another aliquot was treated with 250 mM H_2_O_2_ for 20 min and immediately diluted 10x in an antioxidant solution (0.52 mM citric acid and 10 mM glutation reduced in bidistilled water, pH 7.4). Sperm cryopreservation was carried out following the method described by our group [25]. Briefly, one aliquot was diluted 1∶3 (sperm: extender) in Erdhal & Graham solution (0.7 mM CaCl_2_·H_2_O, 1.08 mM MgCl_2_·6H_2_O, 1.49 mM Na_2_HPO_4_, 34.30 mM KCl, 100 mM NaCl, 0.52 mM citric acid, 55.5 mM glucose, 4.52 mM KOH, 6.48 mM bicine, 323 mOsm/kg, pH 7.4), using 7% dimethyl sulfoxide (DMSO) as permeable cryoprotectant and 10% egg yolk as non-permeable cryoprotectant. Another aliquot was diluted in the same way but the egg yolk was replaced by 12% LDL (Low Density Lipoprotein, extracted from chicken eggs according to Moussa *et al*., [26]). The sperm was equilibrated for 10 min at 4°C, loaded into 1.8 ml cryovials (Thermo Scientific, Denmark) or 0.5 ml straws (Minitube, Germany), respectively, and placed in a horizontal rack 4 cm (cryovials) and 2 cm (straws) above a liquid nitrogen surface in a Styrofoam box for 10 min. The cryovials and straws were then immersed in liquid nitrogen and stored in a nitrogen container until analysis. For thawing, the cryovials were immersed in a water bath (P-selecta, Spain) at 40°C for 2.5 min and the straws at 25°C for 30 s and analysed immediately.

### Fractionation of sperm chromatin

Obtention of the presumably histone and protamine-bound DNA was made following the protocol described by Wykes & Krawetz [27]. The enzymatic digestion steps were performed using *PvuII* as restriction endonuclease and DNA extraction was carried out as it is indicated below. The two fractions obtained, corresponding to the histone- (or histone-like proteins) or protamine-bound DNA, were spectrophotometrically quantified at 260 nm (Nanodrop 1000, Thermo Scientific). Despite of HDNA fraction had not high purity (A_260_/A_280_ <0.5), it was used for the conventional PCR amplification for double checking. For PDNA fraction, only high purity DNA (A_260_/A_280_ >1.8) was used for the PCR assay.

### Extraction of Sperm Nuclear Basic Proteins (SNBPs)

Rainbow trout and seabream (*Sparus aurata*) sperm samples, cryopreserved in straws from our stock, were thawed as described above. Histones from rainbow trout fin and from commercial calf thymus were used as a reference pattern.

The Wykes & Krawetz method [27] was used for the extraction of sperm nuclear basic proteins and somatic histones with some modifications. To obtain histones from rainbow trout fin, a piece of tissue was digested with 1 mg/ml of collagenase for 45 min at 25°C. Somatic cells or spermatozoa were centrifuged at 2000×g for 5 min at 4°C, the pellet was resuspended in PBS and approximately 10^8^ cells were washed twice in 1 ml of TN buffer (25 mM Tris-HCl, pH 8.0; 100 mM NaCl), resuspended in 1 ml of freshly prepared TDTT buffer (50 mM Tris-HCl, pH 8.0; 10 mM dithiothreitol (DTT)) and incubated on ice for 15 min. Following this treatment, 10% (w/v) cetyltrimethylammonium bromide (CTAB) was added to a final concentration of 0.1% and the samples were incubated on ice for 30 min. In sperm samples, the complete removal of tails during this step was examined using differential interference contrast microscopy. After centrifugation at 3000×g for 5 min at 4°C, nuclei were washed twice with TN buffer. Subsequent basic protein extraction was achieved by incubating nuclei with 0.4 N HCl on ice for 1 h (or overnight). After centrifugation at 16000×g for 10 min at 4°C, the supernatant was transferred to a fresh 1.5 ml-microtube and TCA was added to a final concentration of 33% (v/v), keeping the tubes at 4°C overnight in a rotator stirrer (P-selecta, Spain). The tubes were centrifuged at 16000×g for 10 min at 4°C and the pellet was resuspended in 1 ml acetone and kept at −20°C for 4 hours and washed twice with 1 mL of cold acetone. Finally, after centrifugation at 16000×g for 10 min at 4°C, the pellet was dissolved in 50 µl sample buffer (6 M urea; 0.9 N acetic acid; 18.6 mM DTT; 1X protease inhibitor (Complete protease inhibitor cocktail, EDTA-free; Roche Applied Science)) and stored at −80°C.

Protein concentrations were determined using the Bradford method (BioRad, Hercules, CA, USA).

### Acetic acid-Urea Polyacrylamide Gel Electrophoresis (AU-PAGE) and Western blotting analysis

Acidic PAGE was used to create a positive-charged environment in which basic proteins with a high isoelectric point have a net positive charge. Proteins were prepared to reach 1 µg or 5 µg per lane in loading buffer 1X (0.9 M acetic acid, 30% sucrose, 1.2% (w/v) methyl green) with 20 mM DTT. Analysis of SNBPs and the histone pattern was carried out in 20×20 cm gels. Stacking gels consisted on 6 M urea, 0.9 N acetic acid, 6% polyacrylamide, 0.6% N,N,N′,N′-tetramethylethylenediamine (TEMED) and 0.14% ammonium persulfate (APS). Running gel was prepared with 6 M urea, 0.9 N acetic acid, 15% polyacrylamide gels, 0.6% TEMED and 0.14% APS. Electrode leads were switched so that the proteins could run towards the negative end and electrophoresis was set up at 150 V for 7 h. Finally, the gel was stained with Coomassie blue (Biorad, Hercules, CA, USA) for 15 min in a shaker. The gel was washed with a destaining solution of 10% (v/v) methanol and 10% (v/v) acetic acid.

For Western blot analysis, we used the previous conditions with the exception that polyacrylamide gels were 10×8 cm and resolved at 150 V for 80 min. Then, proteins were transferred to a polyvinylidene fluoride (PVDF) membrane (Immobilon-P^SQ^ Membrane 0,2 µm, Millipore, Bedford, USA) using a wet transfer system (BioRad) at 30 V, 90 mA for 16 h. Transfer of proteins was confirmed by staining the blots with Ponceau S solution (0.1% (w/v) Ponceau S in 5% (v/v) acetic acid). PVDF membrane was blocked in 3% non-fat milk, 0.2% Tween-20 in Tris-buffered saline (TBS-T) for at least 1 h at room temperature and incubated overnight at 4°C, with the primary antibody: a rabbit polyclonal antibody against Histone H3 (dilution 1/8000) (ab1791, Abcam, Cambridge, UK). Primary antibody was labeled with secondary anti-rabbit antibody conjugated with horseradish peroxidase (Invitrogen, Camarillo, CA, USA), using a 1/10000 dilution and was developed using the Pierce ECL substrate (Thermo Scientific, Rockford, iL, USA).

### Genomic DNA extraction

Genomic DNA extraction was carried out following the optimized protocol by our group [19]. 10^8^–10^9^ cells from control, UV irradiated, H_2_O_2_ treated and cryopreserved samples were resuspended to a total volume of 700 µl of extraction buffer (10 mM Tris-HCl, pH 8.0; 100 mM ethylenediaminetetraacetic acid (EDTA), pH 8.0; 0.5% (v/v) sodium dodecyl sulfate (SDS)), supplemented with 0.5 µl proteinase K (1 mg/ml). The samples were incubated overnight at 56°C in a shaking temperature-controlled bath (P-selecta, Spain). To eliminate cell components, one volume (700 µl) of phenol-chloroform-isoamyl alcohol mixture (25∶24∶1) was added to each sample. After shaking vigorously for 5 min and centrifuging at 12000×g for 5 min at 4°C, the aqueous phase was immediately collected. This step was repeated twice. Then, the aqueous phase was washed twice with chloroform and DNA precipitation was carried out using absolute ethanol (Scharlau, Spain). Finally, the DNA pellet was resuspended in 30 µl of TE buffer (1 M Tris-HCl, pH 8.0; 0.5 M EDTA) following the indications of Cartón *et al*., [19].

DNA quantity and purity were determined spectrophotometrically at 260 nm (Nanodrop 1000, Thermo Scientific). Only high purity DNA (A_260_/A_280_ >1.8) was used for the qPCR assays.

### Conventional PCR conditions

The amplification of specific primers in the presumably histone or protamine-bound DNA was assessed by PCR in TGradient Thermocycler (Biometra, Goettingen, Germany). Each reaction (20 µl) contained 200 ng of genomic DNA, 0.25 µM of each primer, 6 mM MgCl_2_, 800 µM of dNTPs, 1X PCR buffer MgCl_2_ free (Biotools, Madrid, Spain), 1 U of thermostable DNA polymerase from *Thermus sp*.(Biotools, Madrid, Spain) and up to 20 µl of bi-distilled water. The annealing temperature was 65°C for long oligonucleotides and the extension time was 30 s. Each product size was confirmed by 1.8% (w/v) agarose gel electrophoresis.

### Quantitative PCR

Quantitative real-time PCR of genomic DNA was performed in triplicate using specific primers and non-template control for each pair of primers. Two amplicons with different length, located within the same gene are required to determine DNA damage according to the indications of Rothfuss *et al*., [10]. Oligonucleotide primers were designed using Primer Express 2.0 software (Applied Biosystems, Foster City, CA, USA). Sequences, corresponding to GenBank accession numbers and PCR efficiency are shown in Table 1. Real-time PCR was performed on a Step-One Plus (Applied Biosystems, Foster City, CA, USA) real-time thermal cycler. DNA damage was studied in nine nuclear genes, some of them located in different linkage groups according to Moghadam *et al*., [28]: *HoxA3a-1*, *HoxB5bi*, *HoxC4a-2*, *HoxD4ai*, *HoxD9aii*, *Sox2*, *Eif1b*, *18S rRNA* (18S Ribosomal RNA), *28S rRNA* (28S Ribosomal RNA).

**Table 1:** List of forward and reverse oligonucleotides used in qPCR assay.

Genes and GenBank access. n°.	Forward oligonucleotide	Reverse oligonucleotide	PCR product size (bp)	PCR efficiency (%)
*HoxA3a-1*	CAACCTGCTCAACCTCACG	GTTTGGATCGCACACTCTTTG	571	97.1
AY567795	ATATCCCCCTCCATTGAACAG	CCAGTCCCGGGATACCTCT	69	98.3
*HoxB5bi*	CTCTGAGTCCGAGGAAGGTG	CATCAGGTCCAGCCATTTCT	683	88.5
AY567802	TATTTCCCCTATGTGTTGTCC	ATCAGGTCCAGCCATTTCTAA	69	109.4
*HoxC4a-2*	AACAGCTACATCCCCGACCACAG	TCGCGCACATAGGCTACATAACAG	643	100.8
AY567804	GTGCCTCTAACTCCCATCTCC	CAAAAGCTTCTCCCCTATCGT	57	105.6
*HoxD4ai*	TGTGCAGGGTTCTACAGTGC	TGAGCCAATTAGGTCCCAGT	615	109.0
AY567814	TGTCTATGTGGCCGTCTCAG	AAAACATAGTCATAAGGCAAGTGG	58	98.0
*HoxD9aii*	GCCGCAGTATCAGGGATTTA	AGTTTGCTGCAGGGTTGTCT	687	100.7
AY567817	CCCTGGTTATGCTTGTGGAT	ATCACTGCCAACGCTCTCTT	67	96.6
*Sox2*	AGTTGTCAAGGCTCTGGCGA	GCCTCCCCCTACACCCACT	651	91.9
NM_001141718	ATGGGTTCGGTGGTCAAGTC	GGAGTGAGACGACGACGTGA	66	129,5
*Eif1b*	CCCAGAGTATGGGGAAGTGA	GTTGGTAGCCCAGCATCAAT	634	96.0
NM_001165193	GGCTGCATACGTCCATGTTA	GGCTGCGATGATCAGAACTT	56	91.9
*rRNA 18S*	CCGCAGCTAGGAATAATGGA	CTCAATCTCGTGTGGCTGAA	632	115.5
FJ710873	ATGGCCGTTCTTAGTTGGTG	CCGGAGTCTCGTTCGTTATC	63	91.6
*rRNA 28S*	CGAGATTCCCACTGTCCCTA	ACGCTTGGTGAATTCTGCTT	606	94.3
U34341	GCCTCACGATCCTTCTGACT	CAAGCCAGTTATCCCTGTGG	75	95.9

Genes and their corresponding GenBank accession number are indicated, in addition to the size of the product and oligonucleotide efficiency. All primers are given in the 5′ direction.

Each pair of primers was assayed by conventional and real-time PCR in order to determine optimal conditions for the experiment. Each product size was visualized by agarose gel electrophoresis (data not shown). Reaction conditions were different for long and short fragments, so assays were carried out in different 96-well plates (Applied Biosystems, Spain), using control treatment in each plate. For long amplicons, the reaction mixture contained 4 µl of 5× Fast Start DNA Master plus SYBR Green I (Roche, Germany), 1 µl of each 5 µM forward and reverse primer, 0.4 µl of 50× ROX passive reference dye (BioRad, Foster City), template gDNA (3 ng, except 6 ng for the *Sox2* gene assay) and sterile bidistilled water up to 20 µl. In this case, the reaction conditions were a pre-incubation phase of 10 min at 95°C, followed by 50 cycles of 15 s at 95°C, 10 s at the annealing temperature of 65°C (63°C for *rRNA 18S*) and 50 s at 72°C. For short fragments, the reaction mixture consisted of 10 µl of 2× SYBR Green PCR (Applied Biosystems, Spain), 1 µl of each 5 µM forward and reverse primer, 3 ng of template DNA (6 ng for *Sox2*) and bidistilled water up to 20 µl. PCR reaction began with a pre-incubation phase of 10 min at 95°C, followed by 50 cycles of 15 s at 95°C and 1 min at the annealing temperature of 63°C. Product specificity was verified by agarose gel electrophoresis and threshold cycles (C_t_s) were measured by StepOnePlus version 2.2.2 (Applied Biosystems).

Primer efficiencies were determined using serial dilutions of gDNA (up to 1∶1000000 for all oligonucleotides, except for short amplicons corresponding to *HoxA3a-1*, *HoxB5bi*, *HoxC4a-2* and *Sox2* whose serial dilutions were up to 1∶729), corresponding to ∼1000 ng/µl to 0.01 ng/µl of DNA and ∼180 ng/µl to 0.74 ng/µl, respectively. The amplification was made with long and short fragments, employing the same above-mentioned conditions. PCR efficiencies were calculated with StepOnePlus version 2.2.2 (Applied Biosystems) using the linear regression slope of the dilution series (Table 1).

### DNA lesions rate analysis

The number of DNA lesions per 10 kb was calculated according to the formula [10]: 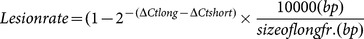



Differences between the C_t_ values of treated samples (UV irradiated, hydrogen peroxide treated and cryopreserved) and non-treated samples were determined for each long and short amplicon. The analysis was done in samples from each male independently, obtaining the number of lesions promoted by the different damaging agents per 10 kb of DNA, respect to the basal level of lesions in untreated samples. Mean ± SEM (standar error of the mean) of lesions from the 7 males were calculated.

Moreover, DNA damage was determined by calculating amplification efficiency (AE) [29]: 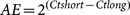



This value was expressed as an amplification efficiency percentage (AE%) relative to the untreated sperm.

### Detection of 8-hydroxyguanosine (8-OHdG) by immunofluorescence

Non-treated and treated sperm samples were washed once in PBS and subsequently diluted to a final concentration of 5·10^6^ cells/ml. Spermatozoa were fixed using 4% paraformaldehyde in PBS for 20 min at room temperature and washed three times with bi-distilled water. Next, 20 µl droplets of spermatozoa were dripped on ATE ([3-aminopropyl]trimethoxysilane) coated slides and desiccate at 37°C overnight. For the rest of the protocol we followed the indications of Koubek P. *et al.,* [30] with few modifications. Nuclei were permeabilized using 30 µl of a solution 0.3% Triton-X 100 in PBS, for 5 min at 4°C. After permeabilization, slides were washed twice with PBS during 10 s, each sperm spot was treated for 1 h at 4°C with 30 µl of blocking solution (20% goat serum (Gibco, New Zeland)) in PBS and washed in PBS for 10 s. The presence of 8-OHdG was revealed by a mouse monoclonal antibody (ab62623, Abcam, Cambrigde, UK) at a dilution of 1/1000 incubating at 37°C for 1 h. Following three washing steps with PBS during 10 s, spermatozoa were incubated with a goat anti-mouse secondary antibody labelled with fluorescent orange-red Alexa Fluor 568 (Invitrogen, Camarillo, CA, USA) at 37°C for 1 h. Slides were washed three times with PBS, stained with DAPI and subsequently mounted using the VECTASHIELD Mounting Media for Fluorescence (Vector Laboratories, Peterborough, UK) for observation using a Nikon Eclipse TE-2000 confocal microscope (Nikon Instruments, Melville, New York, USA).

### Statistical analysis

Statistical analysis was performed with SPSS version 21.0 (IBM, EEUU). A non parametric Kruskal-Wallis test was performed and significant differences were detected using the Dunn-Bonferronipost hoc test (p <0.05). Results are shown as media ± SEM.

## Results

### Histones are totally replaced by protamines in trout sperm

**Figure 1:**
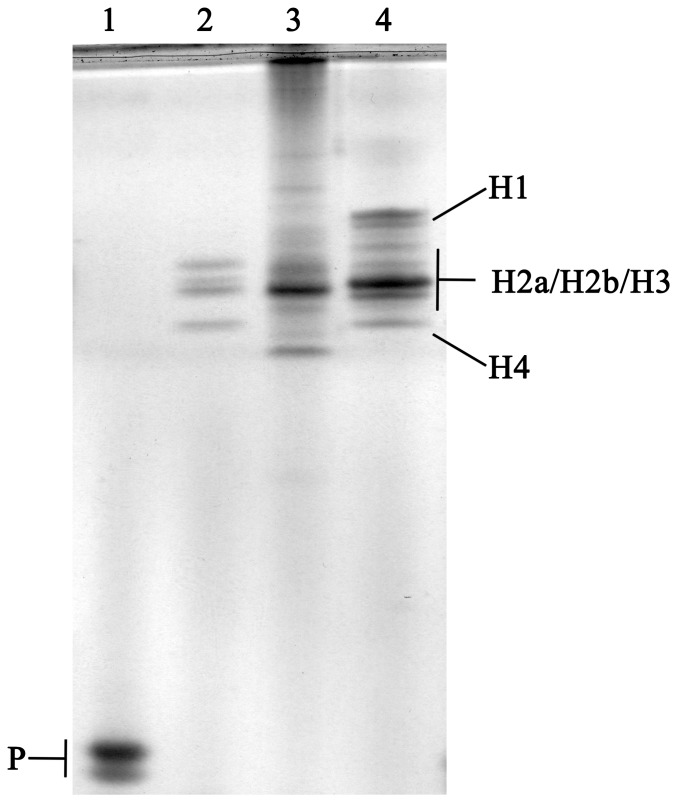
Acetic acid (5%)-urea (6 M) (AU)-polyacrylamide gel electrophoresis (PAGE) analysis of sperm nuclear basic proteins (SNBPs). 1.- *Oncorhynchus mykiss* sperm (1 µg per lane); 2.- *Sparus aurata* sperm (1 µg per lane); 3.- *Oncorhynchus mykiss* fin (5 µg per lane); 4- Commercial histones from calf thymus (5 µg per lane). H denotes “histone-type proteins” and P “protamine-type proteins”.

**Figure 2:**
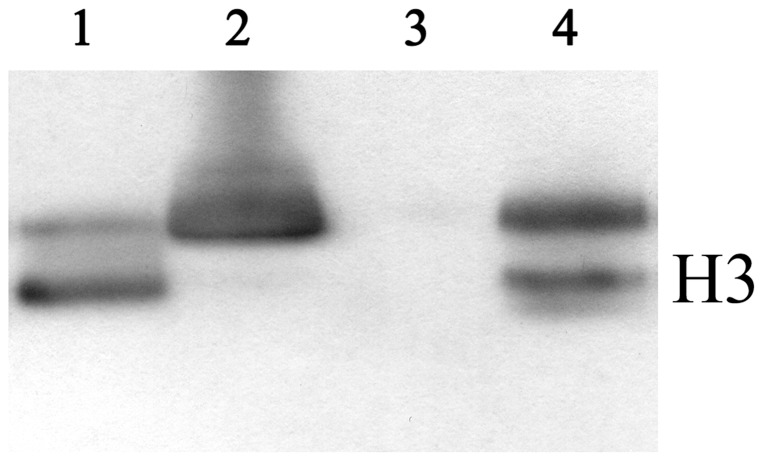
Western blot analysis of sperm nuclear basic proteins (SNBPs). Histones from rainbow trout fin (lane 1), SNBPs from seabream (lane 2) or rainbow trout and histones from commercial calf-thymus (lane 4) were subjected to Western blot (10 µg per lane) with anti-histone H3 antibody.

**Figure 3:**
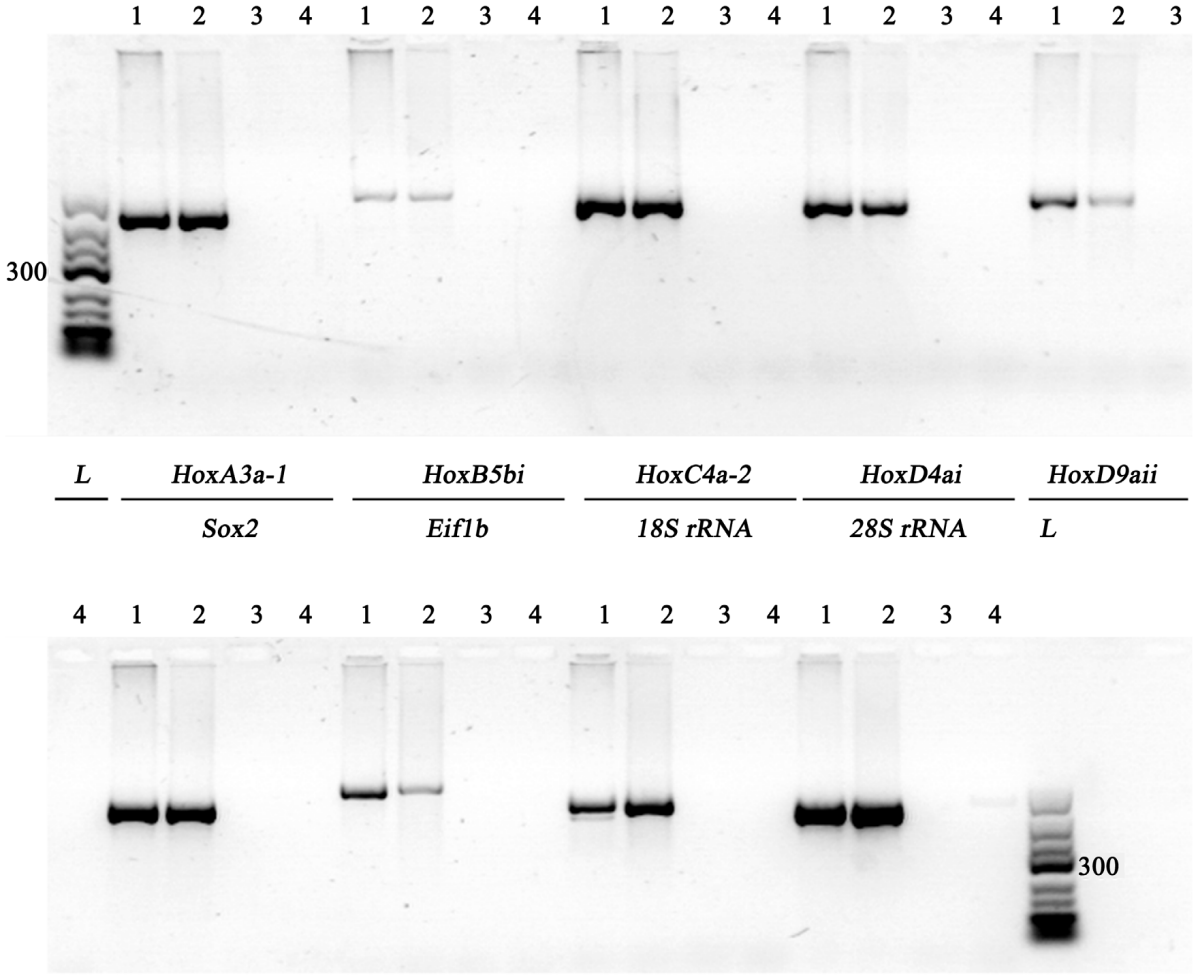
Amplification of nine nuclear genes after DNA fractionation. Electrophoresis analysis of PCR products in genomic DNA (1), digestion resistant DNA (PDNA) (2)digestion sensitive DNA(HDNA-like) (3) and negative control containing no DNA (4). 3 µl of PCR reaction was subjected to agarose (1.8% w/v) gel electrophoresis.

The electrophoretic pattern in polyacrylamide gels showed the presence of a protein component in trout spermatozoa with a high velocity of migration, corresponding presumably to protamine type SNBPs (Fig. 1, lane 1). No traces of histone type SNBPs, similar to any of the used controls (Fig. 1, lanes 2, 3 and 4 for seabream sperm, histones from rainbow trout fin and commercial histones from calf-thymus, respectively) were noticed. This observation was confirmed by Western blot analysis, which revealed the absence of H3 in rainbow trout sperm (Fig. 2, lane 3). Histones from rainbow trout fin, SNBPs of seabream, whose chromatin sperm is compacted by histones and histones commercial pattern were used as control of antibody binding (Fig. 2, lanes 1, 2 and 4, respectively). Additionally, the separation of sperm chromatin sensitive to digestion rendered a HDNA-like fraction under 50 ng/µl with A_260_/A_280_ as low as <0.5 and a resistant PDNA-like fraction of 500 ng/µl approximately with high purity. The studied genes only amplified showing the expected band in genomic DNA and PDNA fractions with no traces in HDNA-like fraction (Fig. 3).

### Not all of the studied genes are equally sensitive to damage

Damage generated by UV irradiation promoted a similar number of lesions in all of the analyzed genes, ranging from 11.42±1.2 for *28S* to 13.29±0.8 for *HoxD4ai* (expressed as the number of lesions per 10 kb ± SEM). This data is in accordance with AE values, being 24% for the former and 14% for the latter, relative to fresh sperm.

**Figure 4:**
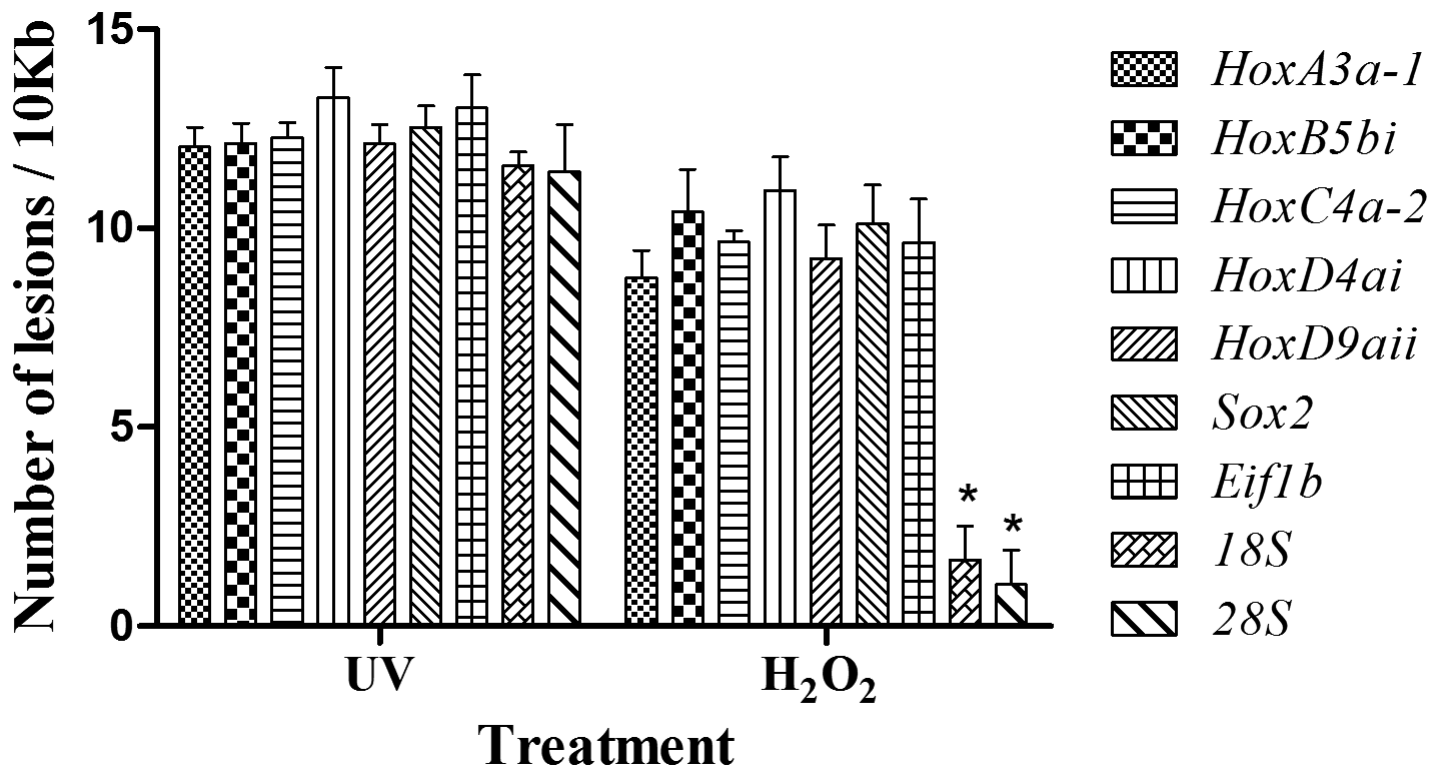
Number of DNA lesions per 10 kb in nine nuclear genes. UV irradiation (400 µW/cm^2^, 10 min) and H_2_O_2_ treatment (250 mM, 20 min) induces different levels of DNA damage in early or late transcribed genes. DNA damage was calculated by the 2^-ΔΔCt^ method and transformed into a DNA lesions rate. Data are expressed as media ± SEM (n = 7). Asterisks show significant differences among genes for the same treatment (*P*<0.05).

However, oxidative stress promoted by H_2_O_2_ caused a significantly lower number of lesions in rDNA genes *18S* and *28S* (1.67±0.8 and 1.05±0.9, respectively), than in the rest of the analyzed genes (ranging from 8.76±0.7 for *HoxA3a-1* to 10.95±0.9 for *HoxD4ai*, expressed as lesions per 10 kb ± SEM) (Fig. 4).

### Different genotoxic agents could alter specific regions in the nucleus

**Figure 5:**
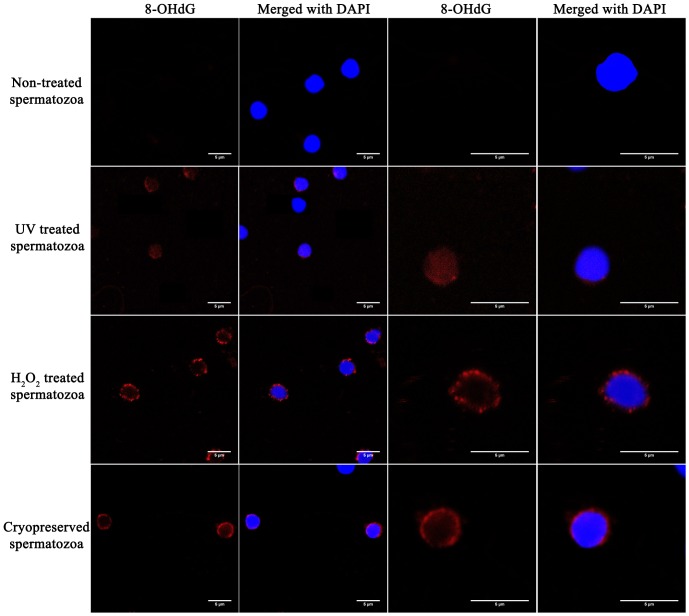
8-OHdG localization in specific nuclear regions in rainbow trout spermatozoa. Representative confocal images (4X and the corresponding 10X screen magnification) from spermatozoa showing the localization of 8-OHdG, labelled with AlexaFluor568, showing the oxidative lesion in red. Cell nuclei were stained with DAPI and appear blue in color. Scale bar, 5 µm.

The nuclear distribution of 8-OHdG was dependent on the genotoxic treatment applied to the sperm. In untreated sperm, no fluorescence was detected; although in UV-irradiated spermatozoa, 8-OHdG display a homogeneous and low intensity pattern of labelling distributed throughout the sperm nucleus. In sharp contrast, cells treated with hydrogen peroxide showed an intense labelling in the peripheral nuclear region with no traces of 8-OHdG in the central area (Fig. 5).

### Effect of cryopreservation in developmental-related genes

**Figure 6:**
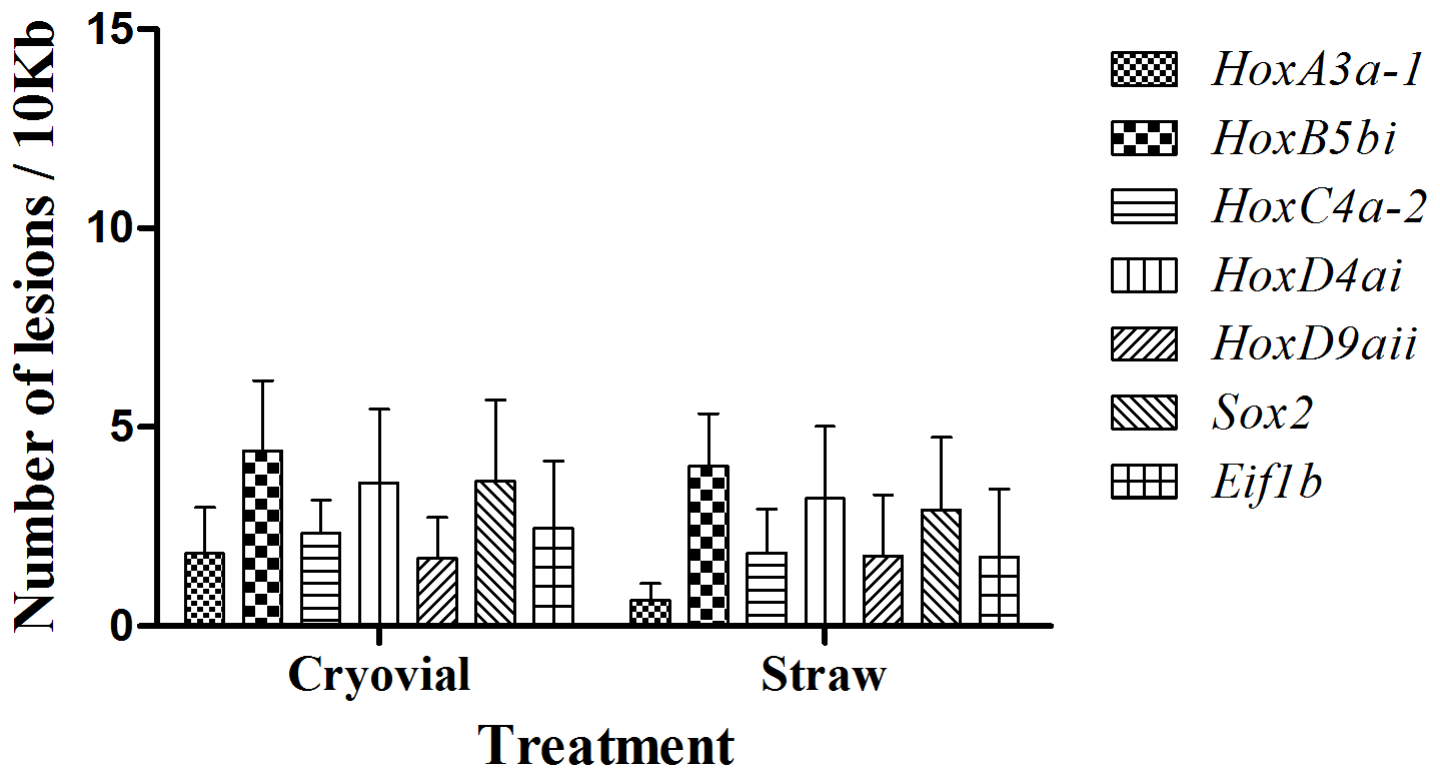
Number of DNA lesions per 10 kb in developmental-related genes. No significant differences are observed after cryopreservation in the studied genes. DNA damage was calculated by the 2^-ΔΔCt^ method and transformed into a DNA lesions rate. Data was expressed as media ± SEM (n = 7). Letters show differences among genes for the same treatment (*P*<0.05).

The number of lesions produced by the two different cryopreservation protocols was always lower than that produced by UV irradiation or oxidative stress treatment. No differences between freezing methods were observed in the number of lesions in any of the developmental-related genes. However, not a substantial susceptibility to damage among them was noticed. As shown in Fig. 6, *HoxA3a-1* suffered lower damage after the cryopreservation process: 1.83±1.2 lesions per 10 kb freezing in a cryovial and 0.65±0.4 lesions per 10 kb of DNA freezing in a straw, corresponding to 90% and 100% of AE relative to untreated sperm respectively. *HoxB5bi* is prone to have higher number of lesions after cryopreservation: 4.40±1.8 lesions for the cryovial and 4.04±1.3 for the straw, with 38.2% and 51.8% of AE, respectively. The distribution of the oxidative lesions are mainly peripheral, more similar to those promoted by H_2_O_2_ than the ones caused by UV irradiation (Fig. 5).

## Discussion

The discovery of different structural elements in the mammalian sperm chromatin -related to the association to different basic nuclear proteins [4], [5]- is the basis for the hypothesis of a different susceptibility to damage among genes. The confirmation of this hypothesis could support a candidate gene approach to selecting genotoxicity biomarkers between those genes particularly sensitive to damage. However, our results showed that sperm nuclear basic proteins are not responsible for different packaging in trout, where, as was also reported in keta salmon [15], all the histones were substituted by protamines. Similar results were obtained by Saperas *et al*., [18] but not by Avramova *et al.*, [24], who reported the presence of histones-type proteins in rainbow trout sperm. Our study demonstrated that histone H3 are absent in trout sperm and all the studied genes locate in chromatin regions similarly resistant to enzymatic digestion, discarding the possible differential packaging by SNBPs similar to that occurring in mammals. Sperm from other fish species, such as zebrafish [7] and seabream [17], is homogeneously compacted with histones. Nevertheless in spite of the apparently homogeneous pattern of compaction, different chromatin packages have also been described in zebrafish according to DNA and histone methylation patterns [7]. These epigenetic marks display a distinctive pattern of DNA hypo-methylation and specific histone modifications (H3K4me3 and H3K14ac) in genes that are important for embryo development [7], those that would presumably be located in histone-bound regions in mammals. This fact reveals that factors different from the retention of histone-bound DNA in specific genes should drive accessibility for early embryo transcription in fish. The replacement of histones by protamines has always been considered a mechanism to increase protection against DNA damage, but differential epigenetic marks could also confer a different degree of protection. This hypothesis requires confirmation. The comparison between species with different chromatin compacting models will allow us to figure out (i) whether gene groups are presented with different levels of accessibility in sperm chromatin and (ii) whether these genes have a different degree of protection against genotoxic agents.

The analyzed genes were selected considering their belonging to the group of genes packaged in structural blocks related to early embryo development (*HoxA3a-1*, *HoxB5bi*, *HoxC4a-2*, *HoxD4ai*, *HoxD9aii*, *Sox2* and *Eif1b*) or their absence from these specific packaging blocks (*18S* and *28S*). The availability of genomic annotations long enough to design pairs of primers giving amplicons longer than 600 ppb was a prerequisite. According to data provided by Wu *et al*., [7], *sox2* and *eif1b* have been identified in zebrafish amongst the top 250 genes enriched in histone marks specific for early expressed genes. *eif1b* is expressed since 2 h post-fertilization (hpf) and *sox2* at 6 hpf. *hoxA3a*, expressed 6 hpf, is together with *hoxC4a*, *hoxD4a* and *hoxD9a*, among the 250 genes with a lower degree of DNA methylation in zebrafish sperm. Moreover *hoxa3a* and *hoxb5b* are among the 250 most enriched genes in some specific histone modifications [7]. Nevertheless, no similar features were identified in *18S* or *28S* genes, whose transcripts have been detected in spermatozoa [7]. These data support the choice of genes, pointing to a differential packaging or epigenetic marks in the two sets of genes. The method applied for the analysis of the number of lesions considers the delay promoted by DNA lesions in the amplification of a long DNA fragment, relative to the amplification of a short fragment of the same gene [10]. This method, based on relative and not on absolute measures, allow the comparison between genes independently that they are single (*HoxA3a-1*, *HoxB5bi*, *HoxC4a-2*, *HoxD4ai*, *HoxD9aii*, *Sox2* and *Eif1b*) or multiple (*18S* and *28S*) copy genes. Moreover, the use of different aliquots from the same male to perform all the treatments allowed us to develop the analysis male by male, considering the amplification in the untreated sperm as the basal level of damage in each particular individual, the basis of the Rothfuss methodology [10]. The used sperm was from the same sources and conditions that those samples evaluated in previous studies for DNA fragmentation using the comet assay [31] giving values lower than 6,5% of tail DNA in untreated samples.

Among the genotoxic treatments applied, UV irradiation is usually used in fish reproduction for inactivating spermatozoa genome in gynogenetic procedures, at higher intensities and irradiation times than those applied in our study. Dietrich *et al*., [32] showed how UV irradiation at a much higher intensity than that used in our study caused an enormous increase in DNA fragmentation in trout spermatozoa after 5 min exposure. Moreover, they observed that most of the embryos produced by UV-irradiated sperm did not reach hatching. It is well-known that UV activates different photosensitizers located both in the nucleus and the cytoplasm [33]. These photosensitizers have the ability to induce DNA fragmentation, the formation of cyclobutane pyrimidine dimer photoproducts (CPDs) and, to a lesser extent, bases oxidization [34]. The oxidization of nucleotides, mainly the generation of 8OHdG, takes place following two different pathways types: Type 1 involves a direct electron transfer from guanine to and excited nuclear photosensitizer without any intermediate; whereas in Type II the energy is transferred to molecular oxygen, generating ROS, which subsequently oxidize the nucleotide[35], [36].

Hydrogen peroxide is one of the most important reactive oxygen species (ROS). Exogenous H_2_O_2_, frequently used as positive control of DNA fragmentation[19], [31], [37], enters into the cell thorough specific aquaporins, the peroxiporins [38], where is metabolized by cytoplasmic and mitochondrial enzymes producing hydroxil radicals [39], [40], having a wide range of cytotoxic effects. Reaching the nuclear region can also produce hydroxyl radicals via Fenton reaction by the presence of transition metal ions associated to DNA [39]. The promoted free radicals oxidize nitrogenous bases [41], being 8-OHdG the most predominant oxidation [42], [43].

The genotoxic effects of these two agents are, therefore, different and provide different results. UV causes a similar number of lesions in all the analyzed genes, suggesting their ability to penetrate and to directly affect the whole genome regardless of the potential differences in chromatin modifications. The differential epigenetic pattern among the analyzed genes did not make any of them more resistant to the irradiation. The homogeneous distribution of 8-OHdG within the nucleus also supports this idea. Nevertheless, the significant differences observed between genes after H_2_O_2_ treatment, reveal that *18S* and *28S* genes are significantly more resistant to oxidative stress than the key genes for embryo development. Moreover the immunodetection of 8-OHdG clearly point out to a positional effect, suggesting a much more intense exposure to the oxidative stress of genes localized at the peripheral area of the nucleus. This point supports the hypothesis of differential susceptibility to damage amongst genes, but surprisingly reveals that it is dependent on the source of damage. Differences in susceptibility to H_2_O_2_ could be explained as a consequence of different localization within the chromosome, differences in their attachment to the sperm nuclear matrix or to the different epigenetic status that could create steric impediments, hindering ROS access to the less accessible and more protected genes. Nevertheless this is not supported by the UV results. The genes most susceptible to oxidative stress promoted by H_2_O_2_ matched with developmental-related genes suggesting a peripheral distribution for all of them and a more central localization for ribosomal genes. The peripheral localization of 8-OHdG was also noticed by Noblanc *et al.,* [9] in sperm from gpx5^-^/^-^ mouse mutants. These authors colocalized the modified guanine with the histone H3 and the nuclear matrix protein TOPO2β, concluding that the association of genes to these proteins renders them more vulnerable to oxidative stress. Our results indicate that, in rainbow trout, the peripheral distribution of damage is independent on the nuclear basic proteins. According to our results differential susceptibility is driven by the accessibility of the genotoxicant to the compacted nuclei, being the central chromatin more protected against H_2_O_2_ with a limited capacity to penetrate across the chromatin (not the case for UV). Peripheral distribution of developmental-related genes suggested by our results could have relationship with their early accessibility to the transcription machinery after fertilization. The structural organization of the chromatin in specific blocks (nuclear basic proteins and other epigenetic marks) has been related to early accessibility to the transcription machinery after fertilization [5], [6], but the positional factor seems to have a predominant role in the differential sensitivity to DNA damage in the case of damages promoted by ROS originated in the cytoplasm.

It is well known that cryopreservation promotes DNA fragmentation and most authors assume that this effect is dependent on the generation of ROS during freezing procedures, leading to the formation of 8-oxodG, 8-oxodA, abasic sites or thymine dimers, among others [31], [41], [44]. Trout sperm suffer DNA strand breaks and bases oxidization after freezing with the procedures applied in this study, as shown by Perez-Cerezales *et al.,* [45] using the comet assay. Considering the differential susceptibility to oxidative stress, the analysis of sensitive developmental-related genes instead the traditional analysis of global genome fragmentation, should be much more sensitive and revealing. The analysis of damage in genes sensitive to oxidative stress indicated slight and non-significant differences in susceptibility to cryodamage among them, without differences between the two freezing protocols and the immunodetection of 8-OHdG also revealed a peripheral distribution of oxidative damage, as could be expected. All the *Hox* genes analyzed are located in different clusters, except the two *HoxD* genes, which in spite of being located in the same cluster, belong to different linkage groups [28]. Their degree of DNA methylation and histone modifications, even if similar, is not identical and their transcription is not synchronized. According to studies by Thummel *et al*., [46], who analyzed the expression of *hoxc13* orthologs in zebrafish, some hox mRNAs are maternally inherited, whereas others are expressed in the embryo at different developmental stages. Moreover, it is well known that *Hox* genes show collinearity: spatial and sometimes temporal ordering of expression, corresponding to their 3′ to 5′ genomic order in a *Hox* cluster [47]. Variability in the pattern of expression, and in the requirements of the paternal gene transcription, could be higher in salmonids, probably the vertebrates with a higher number of clusters and paralogous -13 clusters and 118 *Hox* genes in *Salmo salar* [23], [48]. So we could expect structural and positional differences between them that could affect their cryo-tolerance. This circumstance implies that the selection of the best DNA damage biomarkers should require the analysis of a wide number of candidates. Moreover, gene evolution after genome duplication in fish has been very diverse [21], suggesting that the selection of biomarkers should be specific for groups of close-related species because differences in the timing of expression should imply differences in susceptibility to damage. According to our results the number of lesions promoted by freezing/thawing was much lower than the observed with the genotoxic treatments and more variable between samples. *HoxA3a-1* has an apparent lower sensitivity whereas *HoxB5bi* showed the highest number of lesions. Results suggest that any of the analyzed genes could be good markers of genotoxic damage promoted by oxidization during freezing.

Previous studies by our group reported a lower number of lesions in seabream sperm after cryopreservation [19]. However, we should consider that seabream spermatozoa suffers a lower degree of fragmentation than trout chromatin during cryopreservation, as reported by Cabrita *et al*., [37] using the comet assay. On the other hand, the two nuclear genes analyzed by Cartón-García *et al*., [19], *Igf1* and *Gh*, even if related to growth and development, could be located in more protected regions of the genome. In fact none of them were identified by Wu and colleagues [7] in the chromatin packages with distinctive marks for early transcription in zebrafish sperm.

In conclusion, our study (i) establishes, for the first time, that differences in susceptibility to damage are specific for the genotoxic mechanisms, being observed after oxidative stress but not after UV irradiation; (ii) confirms that positional factors are determinant for the sensitivity to the oxidative damage promoted by H_2_O_2_; (iii) reveals that this fact is not dependent on the nuclear basic proteins associated to DNA in trout and (iv) identifies several peripheral genes as good biomarkers of oxidative genotoxic damage promoted during cryopreservation in trout sperm, allowing early and sensitive detection of genetic damage.
